# Exploring the Effects of Sleep Deprivation on Physical Performance: An EEG Study in the Context of High-Intensity Endurance

**DOI:** 10.1186/s40798-024-00807-4

**Published:** 2025-01-23

**Authors:** Shanguang Zhao, Majed M. Alhumaid, Hai Li, Xin Wei, Steve SHYH-Ching Chen, Hongke Jiang, Yuwu Gong, Yun Gu, Haiquan Qin

**Affiliations:** 1 Department of Physical Education, Shanghai Maritime University, Shanghai, China; 2 Department of Physical Education, College of Education, King Faisal University, Al-Ahsa, Saudi Arabia; 3 College of Sport, Neijiang Normal University, Neijiang, China; 4 School of Humanities and Education, Xi’an Eurasia University, Xi’an, China; 5 Expert Workstation in Sichuan Province, Chengdu Jincheng College, Chengdu, China; 6 Department of Physical Education, Tongji University, Shanghai, 200000 China

**Keywords:** Physical Endurance, Sleep Deprivation, EEG, Power Spectrum, Phase-locking Value

## Abstract

**Background:**

While the effects of sleep deprivation on cognitive function are well-documented, its impact on high-intensity endurance performance and underlying neural mechanisms remains underexplored, especially in the context of search and rescue operations where both physical and mental performance are essential. This study examines the neurophysiological basis of sleep deprivation on high-intensity endurance using electroencephalography (EEG). In this crossover study, twenty firefighters were subjected to both sleep deprivation (SD) and normal sleep conditions, with each participant performing endurance treadmill exercise the following morning after each condition. EEG signals were recorded before and after high-intensity endurance exercise, and power spectrum analysis and functional connectivity analysis were performed on sleep related frequency bands rhythm: δ (0.5–4 Hz) and θ (4–8 Hz). The EEG power spectral and functional connectivity were measured by repeated measure analysis of variance.

**Results:**

The SD condition had an average sleep duration of 3.78 ± 0.69 h, while the duration for normal sleep was 7.63 ± 0.52 h. After high-intensity endurance exercise, the SD condition had a higher maximum heart rate (*p* < 0.05) and shorter exercise time (*p* < 0.05) than normal sleep. Compared with before exercise, the δ band in the left parietal lobe P7 channel increased significantly (*p* < 0.01), and the θ band in the central Cz channel and the left and right parietal lobe P7 and P8 channel increased significantly (*p* < 0.01 & *p* < 0 0.05) in SD and normal sleep conditions after exercise. After exercise, compared with normal sleep, the δ band power in occipital O1 and Oz channels and parietal P7 and TP7 channels in SD significantly decreased (*p* < 0.05 & *p* < 0.01); the power of the θ band decreased significantly in the occipital O1 channel, central CZ channel and the left and right parietal P7 and P8 channel (*p* < 0.05 & *p* < 0.01). Whole connectivity showed a significant increase (*p* = 0.001) in the δ band for the SD condition at post-exhaustion. Local connectivity analysis identified a localized network in the δ band with reduced (*p* < 0.001) post-exhaustion in the SD condition displaying inter-hemispheric differences in certain connections (FP1-CP4, T7-C4, T7-TP8, and O1-FT8) and intra-hemispheric (C3-CPz and Pz-P4) variations.

**Conclusions:**

Sleep deprivation significantly reduced maximum endurance performance, indicating decreased neural activity in the central and parietal brain regions. Alterations in δ and θ frequency band power, along with disrupted connectivity, may highlight the neurophysiological basis underlying this decline.

## Key Points

• Sleep deprivation significantly impairs high-intensity endurance performance, demonstrated by reduced time to exhaustion and increased maximum heart rate compared to normal sleep conditions.

• Sleep-deprived states result in a marked decrease in δ and θ band activity within the central and parietal brain regions, which are essential for motor control and endurance capacity.

• Sleep deprivation diminishes the efficiency of information transmission within the δ band network, suggesting disrupted connectivity that may hinder cognitive and motor processes critical to sustaining performance.

## Background

In emergency and rescue operations, rescuers are required to maintain a high level of physical and mental performance for extended periods in extreme conditions, placing significant demands on their physical and cognitive functions [1, 2]. However, these personnel often face the challenge of sleep deprivation while performing their tasks, a situation that is common among various roles, including firefighters, emergency medical service personnel, and search and rescue team members [3–5]. Research indicates that insufficient sleep negatively impacts cognitive function but also reduces physical performance, resulting in delayed reaction times and diminished muscle strength [6, 7]. Although studies have explored the effects of sleep deprivation (SD) on the general population [8], there is still limited research specifically addressing the neurological impacts on this unique occupational group of rescuers.

Existing research has revealed the widespread negative impacts of SD on physical functioning, including instability in the nervous system and a decline in muscle function [9, 10]. These effects are particularly pronounced during emergency tasks, where the stability of the nervous system is crucial for the responsiveness and coordination of rescuers. Insufficient sleep can disrupt neuronal activity and slow neural transmission, thereby affecting task execution [11, 12]. Furthermore, insufficient sleep significantly reduces muscular endurance and strength, increasing the likelihood of fatigue among rescuers, a point supported by multiple studies. For instance, Curcio and Bradke [13] revealed that a significant decline in sprint performance among soccer players after 30 h of continuous sleep deprivation. Similarly, long-distance runners exhibited reduced running distances, heightened feelings of fatigue, and impaired emotional responses and attention under sleep-deprived conditions [14]. Even mild sleep deprivation can adversely affect athletic performance; studies have shown that healthy adolescents experienced a significant reduction in the duration of aerobic exercise after just 4 h of sleep deprivation, accompanied by a notable increase in heart rate [15]. Additionally, insufficient sleep may negatively affect energy metabolism, leading to unstable blood sugar levels and further compromising the physical endurance of rescuers [16].

However, while the physical impacts of SD have been well-documented, there is a notable gap in understanding its effects on the underlying neural mechanisms involved. The central nervous system operates through the orchestrated interplay of neurons, engaging in spontaneous activity and responding to external stimuli with excitatory and inhibitory processes [17]. Electroencephalography (EEG) captures the electrical dynamics of brain neurons, which manifest as diverse wave forms across different frequency spectra, including δ (0.5–4 Hz), θ (4–8 Hz), α (8–13 Hz), β (13–30 Hz), and γ (30–100 Hz) [18]. Each wave category corresponds to distinct states of brain activity, with α frequency rhythm aligning with relaxation and β frequency rhythm correlating with alertness and cognitive engagement [19, 20]. Sleep-associated brain rhythm predominantly falls within the low-frequency range of slow waves (1–8 Hz) [21, 22].

To investigate the effects of SD on the neurophysiological basis of endurance performance, it is essential to conduct both EEG power spectrum and functional connectivity analyses. Power spectrum analysis quantitatively evaluates the distribution of EEG power across different frequency bands, highlighting how specific patterns of brain activity are affected by sleep deprivation [23]. Functional connectivity assesses the temporal correlations between spatially remote brain regions, offering insights into how sleep deprivation alters the interactions and cooperation between different neural circuits [24]. During sleep deprivation, the interplay between homeostasis and circadian rhythms generates sleep pressure, leading to cognitive disruptions characterized by slowed actions and diminished energy in the δ and θ frequency rhythms [25]. Snipes et al. [22] conducted EEG wave amplitude oscillation monitoring in 18 healthy young subjects exposed to three sleep deprivation models, revealing increased power in the θ frequency band across multiple brain regions, particularly in the frontal lobe cortex. Similarly, Song et al. [26] found that average EEG amplitude in the frontal lobe and central area notably decreased following short-term sleep deprivation, while exhibiting a conspicuous increase in the parietal lobe. These findings suggest that SD compromises cognitive functions in the frontal lobe and other brain regions, leading to heightened activation in the parietal lobe. By integrating functional connectivity and power spectrum analysis, we can better understand how sleep deprivation affects neural dynamics and cognitive performance, ultimately informing strategies to mitigate its detrimental effects.

It is noteworthy that firefighters, as representative professionals in rescue operations, undertake high-risk tasks but also require exceptional physical endurance and rapid response capabilities. Their work, similar to that of other rescue personnel, involves prolonged duty periods, sudden tasks, and factors such as sleep deprivation, all of which place significant stress on both physiological and psychological states [27]. Therefore, this study focuses on firefighters to investigate the effects of sleep deprivation on their endurance performance and the nervous system. We hypothesize that sleep deprivation will significantly affect the neural activity of firefighters, particularly in brain regions associated with endurance, thereby influencing their task performance. By exploring this mechanism, we hope to provide more targeted sleep strategy guidance for rescue personnel to help them maintain optimal performance in complex environments.

## Methods

### Participants

The sample size was calculated using G*Power software (version 3.1). Assuming an expected effect size of d = 0.8 (Cohen’s d), a significance level of α = 0.05, and a statistical power of 0.80, the minimum sample size required for this study was determined to be 20 participants. This ensures adequate power to detect differences between conditions across the repeated measures in a crossover design. The study involved twenty male firefighters, all right-handed, with no history of mental disorders. The participants had a mean age of 26.33 years (SD = 2.55), an average of 4.64 years of rescue experience, a mean body weight of 69.70 kg (SD = 6.75), and a mean height of 174.54 cm (SD = 4.56).

To assess participants’ sleep conditions, this study utilized the Pittsburgh Sleep Quality Index (PSQI). Only individuals with a PSQI score of ≤ 5 were included in the study, ensuring that they had good sleep quality and eliminating potential impacts of factors such as chronic sleep deprivation on the EEG results. By employing this method, we aimed to better control for participants’ sleep status and reduce individual variability that could interfere with the study outcomes. Essential demographic information is presented in Table 1. Inclusion criteria were as follows: (1) a minimum of three years of experience in rescue operations; (2) normal vision, with no color blindness or weakness; (3) absence of sleep-related disorders; and (4) no history of brain injury or conditions affecting cognitive, cardiovascular, mental, or neurological functions. All participants provided informed consent before taking part in the study, which was approved by the Academic Ethics Committee of Shaanxi Normal University, in compliance with the ethical standards outlined in the Declaration of Helsinki (202216017).

**Table 1:** Basic information of participants

Index	(*n* = 20)
Age (years)	26.33 ± 2.55
Sex	Male
Height (cm)	174.54 ± 4.56
Body weight (kg)	69.70 ± 6.75
BMI (kg/m^2^)	22.79 ± 2.11
PSQI	≤ 5

BMI, Body Mass Index; PSQI, Pittsburgh Sleep Quality Index scale

### Experimental Protocol

**Fig. 1:**
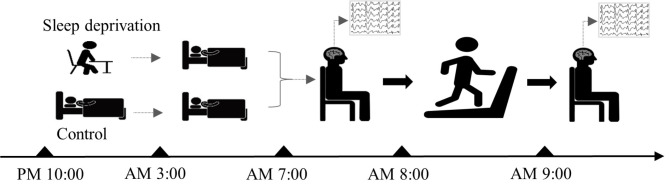
Experimental procedures for sleep deprivation and normal sleep conditions

In this crossover study, participants were subjected to both sleep deprivation and normal sleep conditions, with each participant performing endurance treadmill exercise after each condition. A wash-out period of at least seven days was maintained between interventions to minimize carryover effects. The experimental procedure is shown in Fig. 1. The partial sleep deprivation model defined insufficient sleep as less than 4 h, based on the sleep duration recommendations for adults provided by the American Sleep Foundation. Normal sleep, on the other hand, was defined as at least 7 h of sleep [28].

The sleep model was implemented as follows. At 10:00 pm on the day before the physical exhaustion exercise, the fatigue recovery and exercise performance monitoring system (Bodyguard-2, Firstbeat, Finland) was used to record the sleep duration within 24 h, with a data collection frequency of 1000 Hz. The sleep deprivation (SD) group was instructed to stay awake until 3:00 am and then wake up before 7:00 am, ensuring they slept less than 4 h, thus meeting the criteria for sleep deprivation. In the control group, participants typically fell asleep at 11:00 pm and woke up at 7:00 am, ensuring they slept for at least 7 h. Resting-state data acquisition was completed between 7 and 8 am, and one-time body tasks were completed between 8 and 9 am, followed by another resting-state EEG data acquisition.

### One-time High-intensity Endurance Exercise

The participants underwent a high-intensity endurance exercise on a treadmill (h/p/cosmos cos10253, Germany) at 9:00 am. The Bruce protocol was utilized for the exercise. To ensure participant safety, safety harnesses were securely fastened before starting the treadmill. Participants began at stage 1 of the protocol, which had an initial speed of 2.7 km/h and a 10% incline, as specified in Table 2. Every 3 min, the speed and incline were incrementally increased according to the protocol. During physical activity, participants used a portable blood pressure monitor (Omron, Germany) to monitor changes in blood pressure and heart rate. The rating of perceived exertion (RPE) scale was employed to assess the perceptions of participants at the end of each phase. The stage of the Bruce protocol and the overall exercise duration were promptly recorded upon completion of the exercise.

**Table 2:** Exercise load for all levels of high-intensity endurance exercise

Parameters	Level 1	Level 2	Level 3	Level 4	Level 5	Level 6	Level 7
Speed (km/h)	2.7	4.0	5.4	6.7	8.0	8.8	9.6
Slope (%)	10	12	14	16	18	20	22
Duration (min)	3	3	3	3	3	3	3

The termination criteria followe [29], where participants stopped the exercise if they met any three of the following criteria: (1) Behavioral indications of respiratory distress; (2) Systolic blood pressure exceeding 150 mm Hg or diastolic blood pressure exceeding 75 mm Hg; (3) Heart rate surpassing 180 beats/min; (4) RPE reaching level 18 and encouragement did not result in continuation of exercise.

### EEG Acquisition and Processing

The participants were awakened the next day at 7:00 am to undergo pre-exercise EEG acquisition. One hour later, the endurance exercise was performed, followed by another EEG acquisition 5 min after the exercise concluded. High-resolution EEG collection equipment from Neuroscan, USA, consisting of 32 caps following the International 10–10 method, was used to record EEG signals. The EEG data were captured online, employing a bandpass filter ranging from 0.05 to 100 Hz, and a sampling frequency of 1000 Hz per conductor. Reference electrodes were placed on bilateral mastoids, with forehead grounding utilized. Electrodes positioned above and below the left eye measured vertical electrooculography, while electrodes located on the sides of both eyes measured horizontal electrooculography. The impedance between all electrodes and the scalp was kept below ten kiloohms.

The unprocessed EEG data were pre-processed using EEGLAB, an open-source MATLAB-compatible toolbox. The EEG data underwent filtration using a finite impulse response filter set at a bandpass range of 0.5–45 Hz, and a notch filter at 50 Hz was applied. Subsequently, the EEG waves were segmented into 2-second intervals and transformed into an average reference. An independent component analysis approach was employed to detect and separate artifact components, eliminating portions of the sources containing artifacts such as eye blink artifacts, ocular movements, and electromyography artifacts.

### Power Spectrum Analysis

The EEG signal spectrum for each participant and segment was obtained using a fast Fourier transform (FFT), resulting in a power spectrum (µV^2^) ranging from 1 Hz to 30 Hz. The FFT algorithm is derived from the discrete Fourier transform, which is calculated for a complex sequence *x(n)* with a length of *N* as follows:

$${\text{X}}\left( {\text{k}} \right) = \sum\limits_{s = 0}^{s - 1} {{\text{x}}\left( {\text{n}} \right){{\text{e}}^{ - {\text{j}}\frac{2}{\pi }}}^{{\text{ks}}}\left( {{\text{k}} = {\text{0,1}},2 \ldots \:.{\text{S}} - 1} \right)}$$

*X(k)* represents the data resulting from the DFT to *x(n)*, which represents the sampled analogue signal. The variable *x(n)* in the equation can be a complex signal, while *x(n)* is real with an imaginary component of 0. The formula can be elaborated as follows:

$${\text{X}}\left( {\text{k}} \right) = \sum\limits_{s = 0}^{s - 1} {{\text{x}}\left( {\text{s}} \right)\left( {{\text{cos}}2\pi {\text{k}}\frac{{\text{n}}}{{\text{N}}} - {\text{jsin}}2\pi {\text{k}}\frac{{\text{s}}}{{\text{S}}}} \right)\left( {{\text{k}} = {\text{0,1}},2 \ldots \:.{\text{S}} - 1} \right)}$$

In the equation, *n* represents the s-th sample in the time domain, and *S* represents the signal time series where s ranges from 0 to *s-1*. *X* represents the frequency domain of the time series signal *X*, and *N* represents the k-th sample. Signal *x* represents frequency components, where *K* represents the k-th frequency component with *k* ranging from 0 to s-1. The procedure is executed in MATLAB to obtain the energy values of δ (0.5–4 Hz) and θ (4–8 Hz) frequency bands in the workspace and generates brain topographic maps for each frequency band.

### Phase Locking Value for EEG Connectivity

The study employed the phase locking value (PLV) to investigate the synchronization of phases within the EEG data. This approach can now be used to assess the phase synchronization across multiple channels [30]. The EEG data from all electrodes underwent band-pass filtering to isolate two frequency bands: δ (0.5–4 Hz) and θ (4–8 Hz). The data were processed and transformed into the time-frequency domain using the Hilbert transform to obtain phase details.

The analytical signal of two given signals x(n) and y(n) is as follows:

$$\:\begin{array}{c}x\left(n\right)={x}_{1}\left(n\right)+i{x}_{2}\left(n\right)={a}_{1}\left(n\right){\text{e}}^{\text{i}{{\Phi\:}}_{1}\left(n\right)}\\\:y\left(n\right)={y}_{1}\left(n\right)+i{y}_{2}\left(n\right)={a}_{2}\left(n\right){\text{e}}^{\text{i}{{\Phi\:}}_{2}\left(n\right)}\end{array}$$

where $\:{{\Phi\:}}_{1}\left(n\right)$ and $\:{{\Phi\:}}_{2}\left(n\right)$ represent the instantaneous phase, and $\:{a}_{1}\left(n\right)$ and $\:{a}_{2}\left(n\right)$ represent the instantaneous amplitude of the signals in the two frequency bands, respectively. Then, the PLV can be calculated as:

$${\text{PLV}} = \left| {\frac{1}{N}\sum\limits_1^N {{{\text{e}}^{{\text{i}}\left( {{\Phi _k}\left( n \right) - {\Phi _j}\left( n \right)} \right)}}} } \right|$$

In the equation, *N* represents the total number of trials per participant, while $\phi 1\left( {t,\,n} \right) - \phi 2\left( {t,\,n} \right)$ represents the instantaneous phase difference between channel k and channel j in trial *n* at time t inside the equation. The PLV value ranges between 0 and 1, where PLV is 0 when there is no phase synchronization between the two signals, and the PLV value is 1 when there is complete phase synchronization between the two signals [31]. The statistical results were corrected for multiple comparisons across channels by the false discovery rate (FDR).

### Statistical Analysis

Statistical analysis was performed using SPSS (23.0; SPSS, Inc., Chicago, IL, United States). The Shapiro-Wilk test was used for non-normally distributed data. Paired-sample t-tests were conducted for the behavioral data of participants. For power spectrum values in δ and θ frequency bands, two sleeps (sleep deprivation and normal sleep) × two times (before and after exhaustion) repeated measures analysis of variance (ANOVA) was performed. A Greenhouse–Geisser correction of the ANOVA assumption of sphericity was applied where appropriate. The Bonferroni correction method was used to correct multiple comparisons. The significance level for all statistical tests was set at *p* < 0.05. All figures were generated using MATLAB 2013b and GraphPad Prism 8.0.2.

## Results

### Physiological Parameters Information

The sleep duration of the SD and normal sleep conditions was approximately 3.78 ± 0.69 h and 7.63 ± 0.52 h, respectively. All participants met the criteria for termination of physical exhaustion during the endurance test exercise. Table 3 presents the physiological indicators that signify exercise completion. There were no significant differences in systolic blood pressure and diastolic blood pressure between the SD (SBP: 157 ± 24 mmHg, DBP: 86.28 ± 24 mmHg) and the normal sleep conditions (SBP: 158 ± 17 mmHg, DBP: 75 ± 13 mmHg). However, the SD condition exhibited a significantly higher (*p* < 0.05) maximum heart rate at the point of physical exhaustion, recorded at 194.78 ± 16.12 bpm, compared to the normal sleep condition rate of 183.25 ± 13.36 bpm. Additionally, the SD condition experienced a significantly shorter (*p* < 0.05) duration of exercise, lasting 16.55 ± 1.07 min, compared to 17.15 ± 1.15 min in the normal sleep condition.

**Table 3:** Results of physiological parameters of physical exhaustion

Parameters	Sleep deprivation	Normal sleep
Duration of sleep (h)	3.78 ± 0.69**	7.63 ± 0.52
Duration of exercise (min)	16.55 ± 1.07*	17.15 ± 1.15
SBP (number of > 150 mmHg)	157 ± 24	158 ± 17
DBP (number of > 75 mmHg)	86.28 ± 24	75 ± 13
HR_max_ (number of > 180)	194.78 ± 16.12 *	183.25 ± 13.36

SBP, Systolic blood pressure; DBP, Diastolic blood pressure; HR max, Maximum heart rate. *, *p* < 0.05, **, *p* < 0.01

### Power Spectral Density

**Fig. 2:**
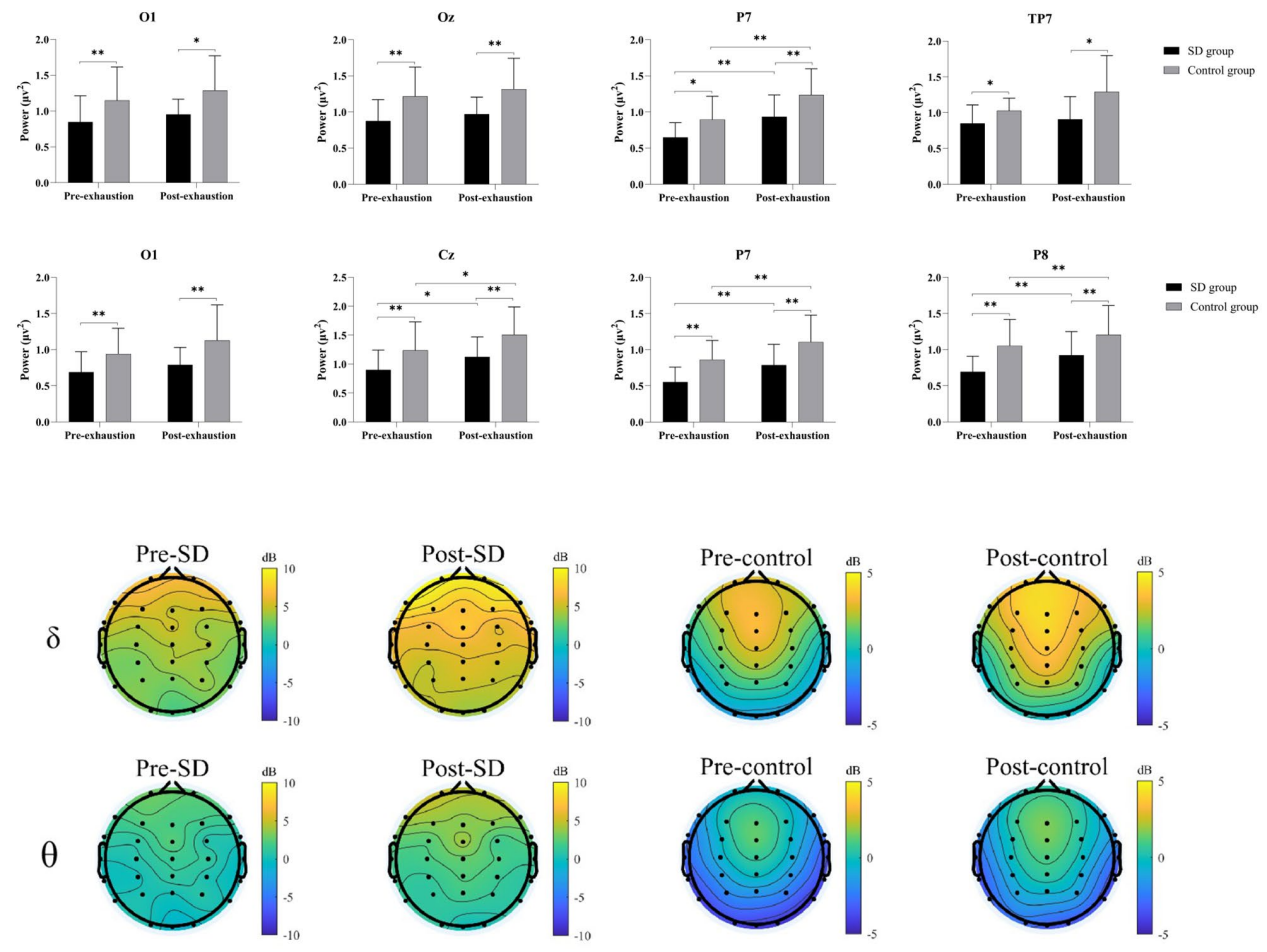
EEG power spectrum and topography results of sleep deprivation and normal sleep conditions SD, Sleep deprivation condition; Control, Normal sleep condition *, *p* < 0.05, **, *p* < 0.01

Figure 2 illustrates the results and displays the topographic map. The results revealed a significant interaction effect between *sleeps × times* for the δ band frequency, F (2, 42) = 1.25, *p* < 0.05, *η* = 0.08. Further analysis indicated a significant reduction in δ band power before exhaustion in the SD condition compared to the normal sleep in channels O1 (*p* < 0.01), Oz (*p* < 0.01), P7 (*p* < 0.05), and TP7 (*p* < 0.05). After exhaustion, the δ band power was significantly lower in the SD condition than in the normal sleep in channels O1 (*p* < 0.05), Oz (*p* < 0.01), P7 (*p* < 0.01), and TP7 (*p* < 0.05). It is worth noting that the δ band in post-exhaustion showed an increasing trend in O1, OZ, and TP7 channels compared to pre-exhaustion in sleep deprivation and normal sleep conditions, although the difference was not statistically significant (*p* > 0.05), except for a significant increase in the P7 channel (*p* < 0.01).

The analysis also revealed a significant interaction effect between *sleeps × times* on θ band power, F (2, 42) = 0.59, *p* < 0.05, *η* = 0.04. The simple effect analysis indicated a significant decrease in θ band power before the onset of exhaustion in channels O1 (*p* < 0.01), CZ (*p* < 0.05), P7 (*p* < 0.01), and P8 (*p* < 0.001) in the SD condition compared to the normal sleep. After exhaustion, there was a significant reduction in θ band power observed in channels O1 (*p* < 0.05), CZ (*p* < 0.01), P7 (*p* < 0.01), and P8 (*p* < 0.05) in the SD condition compared to the normal sleep. Notably, there was no statistically significant difference in the O1 channel before and after exhaustion in either conditions (*p* > 0.05). However, a significant increase was observed in the CZ, P7, and P8 channels following exhaustion (*all p* < 0.05).

### PLV as a Whole Connectivity

**Fig. 3:**
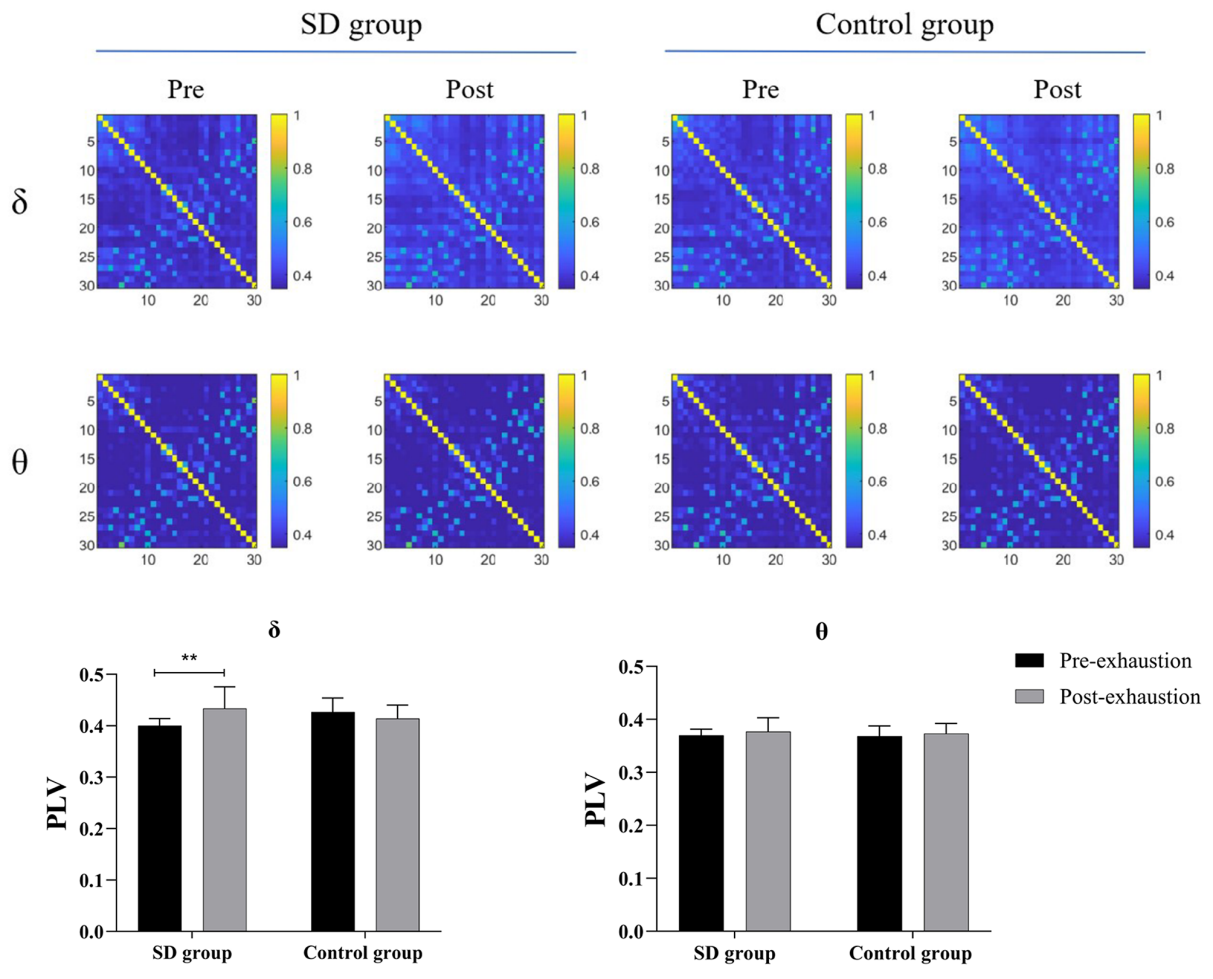
Synchronization matrices across the sleep deprivation and and normal sleep conditions Note: The number of EEG channels is 30, resulting in the 30 × 30 square matrix whose elements represent the average strength of PLV values across the whole subjects between a pair of EEG channels. SD, Sleep deprivation condition; Control, Normal sleep condition

The PLV matrix, presenting grand-averaged values across both the SD and normal conditions for the δ and θ frequency bands, is depicted in Fig. 3. After exhaustion, the mean PLV of the δ band in the SD condition exhibited a significant increase compared to pre-exhaustion (*p* = 0.001). However, there was no significant difference observed in the mean PLV of the δ band between pre- and post-exhaustion phases in the normal sleep group. No significant differences were found within either the SD or the normal sleep conditions for the θ frequency band (*all p* > 0.05).

### PLV as a Local Connectivity

**Fig. 4:**
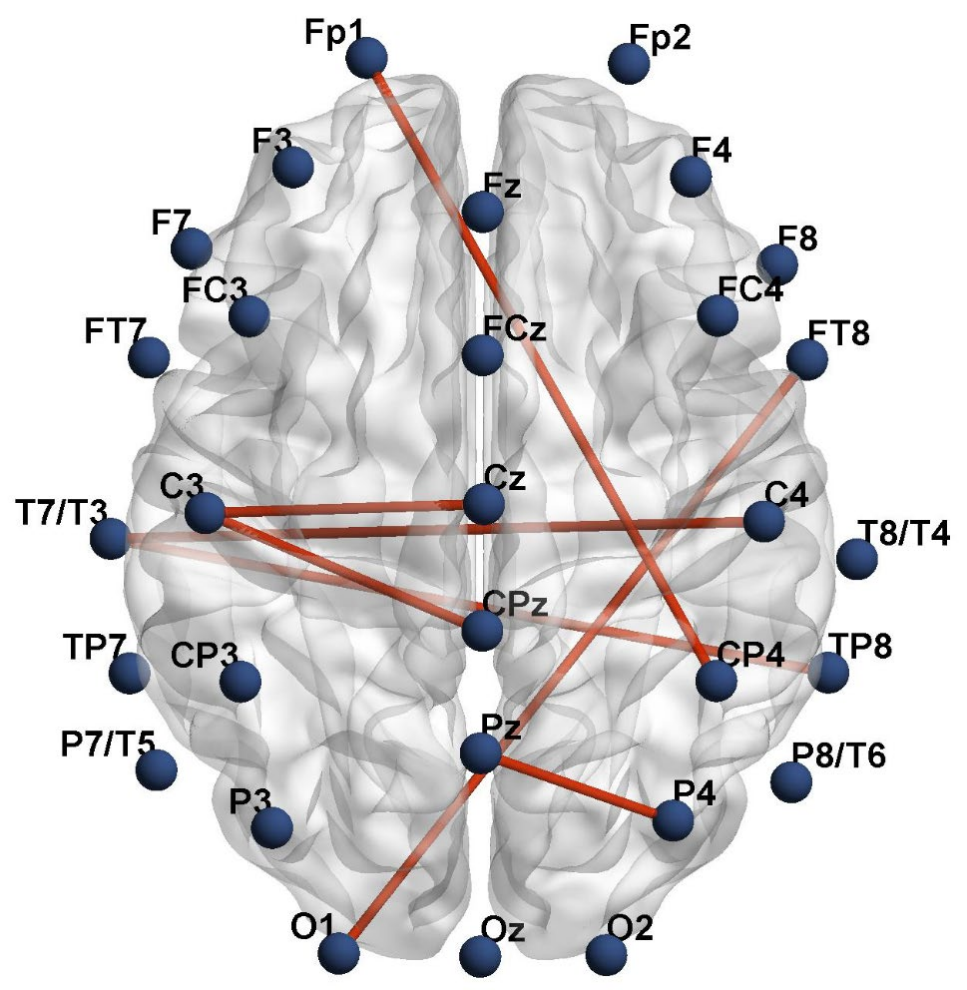
Clustered connections from network-based statistics (NBS) Note: The nodes consisted of FP1, T7, C3, CPz, Pz, P4, O1, TP8, and FT8 and produced increased synchronization in the δ band of the sleep deprivation group compared to the control group (*p* < 0.05, corrected)

Network-based statistics (NBS) analysis identified a single localized network in the δ band with noticeably reduced PLV in the post-exhaustion state compared to the pre-exhaustion state in the SD condition (*p* < 0.001, corrected). The nodes included FP1, T7, C3, CPz, Pz, P4, O1, TP8, and FT8. Inter-hemispheric differences were observed in FP1-CP4, T7-C4, T7-TP8, and O1-FT8 connections, while intra-hemispheric variations were detected in C3-CPz and Pz-P4 connections (Fig. 4).

## Discussion

This study aimed to investigate the effects of SD on physiological and neurophysiological parameters during physical exhaustion in search and rescue personnel. Our findings revealed several key results: first, firefighters in the sleep deprivation condition exhibited significantly shorter exercise durations and higher maximum heart rates compared to those with normal sleep, indicating impaired physical performance under sleep deprivation. Second, power spectral density analysis showed significant reductions in both δ band and θ band powers in the SD condition before and after exhaustion, highlighting the impact of sleep deprivation on brain activity. Lastly, connectivity analysis demonstrated altered local and whole-brain connectivity patterns, further emphasizing the neural consequences of exhaustion associated with sleep deprivation. These findings underscore the importance of sleep in maintaining physical and cognitive functions, suggesting the need for further research into targeted neurointerventions, cognitive training, and sleep hygiene practices to mitigate the negative impacts of sleep deprivation on endurance performance. The insights gained from this research enhance our understanding of the neurophysiological mechanisms involved but also provide a foundation for future studies aimed at optimizing performance in high-stakes environments.

Previous studies have primarily focused on the impact of sleep deprivation on cognitive functions, particularly memory. Nuclear magnetic resonance imaging (MRI) studies have shown that sleep deprivation significantly affects functional connectivity within bilateral hippocampal regions, with increased connectivity observed with brain regions such as the right cingulate cortex, occipital cortex, left frontal gyrus, and temporal gyrus [32]. These regions are closely associated with cognition, memory, and reasoning [33]. In the current study, it was found that both before and after exercise, compared with the normal sleep condition, the power of the δ and θ bands in the sleep-deprived state was reduced in the occipital lobe. The occipital lobe consists of the visual primary cortex and visual association cortex, indicating its involvement in the perception and processing of visual information and complex visual perception processes [34]. Information received by the brain from the incoming visual cortex is transmitted through the occipital lobe to the hippocampus, where it undergoes processing to form a memory [33]. Therefore, it is speculated that sleep deprivation leads to a decrease in neuronal activity in the occipital region, weakening connections to the limbic system (especially the hippocampus), temporal lobe, and parietal lobe. Consequently, the brain’s ability to process information may be impaired, resulting in negative impacts on the memory and cognition of individuals. This physiological mechanism may be associated with sleep deprivation-induced abnormal secretion of various neurotransmitters involved in learning, cognition, and memory in these regions, such as amino acids, cholinergic, peptides, and monoamines [35, 36]. However, it is important to note that no significant difference in power values of the occipital lobe was found between the two sleep conditions after exercise compared to pre-exercise. This suggests that the occipital lobe may not be the primary brain region influencing the performance of high-intensity endurance exercise.

The EEG results demonstrated a significant increase in the power of the δ and θ in channels related to the central and parietal lobes after exercise compared to before exercise, regardless of sleep sufficiency or deprivation. The central area and parietal lobe are involved in the somatosensory processing of the human body and play a crucial movement [37, 38]. Studies have indicated that during fatigue, the movement-related cortex of the brain requires the involvement of neurons from multiple areas to compensate for the decline in function caused by exercise [36]. Jiang et al. [39] used near-infrared spectroscopy to observe changes in brain regions during exercise and found significantly enhanced functional connectivity between the primary motor cortex, premotor area, motor assistance area, and frontal area during fatigue. As exercise-induced fatigue occurs, there is a notable increase in cortical activity within the brain, particularly in the activation and neural interconnection between the motor cortex and various cerebral regions. This surge suggests that the brain engages in heightened neural modulation and synchronization to maintain the motor execution required for muscular control during fatigue. Moreover, these changes involve adjustments in neural network connectivity, leading to increased neuronal oscillations in the motor cortex area. These oscillations may serve as a self-regulating mechanism in the brain to address bodily fatigue and maintain the coherence of motor execution. However, neural adaptations due to fatigue can potentially have a negative impact on motor control, resulting in decreased cognitive functions and motor coordination, consequently limiting endurance and athletic performance. The hypothesis is that within high-intensity endurance activities, the central region and bilateral parietal lobes of the brain may constitute critical core regions influencing endurance performance. With the gradual emergence of exercise-induced fatigue, heightened neuronal activity in the central region and bilateral parietal lobes could potentially be triggered to enhance coordination between muscles and the brain. Activation in these brain regions may contribute to optimizing the muscle-brain axis, improving motor coordination, and potentially prolonging athletic performance sustainability.

We discovered that the duration of sustained high-intensity endurance exercise was significantly shorter in the SD group compared to the control group. However, it is important to note that before exercise, the power of the δ band in the left parietal (P7) was significantly reduced in the sleep-deprived state compared to the sleep-sufficient state. Additionally, the power of the θ band in the central region (CZ) and the parietal lobes (P7 and P8) was significantly decreased. Previous research has indicated that during fatigue induction, the central cortex, responsible for coordinating body movements, inadequately generates neural impulses to sustain motion, necessitating increased activation of neurons in additional cortical regions [39]. Following sleep deprivation, changes in θ wave amplitude oscillation in the central region may be associated with decreased neural activity in the central motor area, leading to reduced transmission of motor signals to the muscles during subsequent high-intensity endurance exercise. As a compensatory effect, the parietal lobes, affected by sleep deprivation, exhibit a decrease in δ and θ neural activity. Consequently, during high-intensity endurance exercise, the significant reduction in functional connectivity between the left and right parietal lobes, which are linked to motor control-related cortical areas, results in premature fatigue among sleep-deprived individuals, impairing subsequent high-intensity endurance performance. Previous research suggests that sleep deprivation may lead to alterations in the microstructural composition of the central and parietal lobes of the brain. Three specific studies support this claim. Firstly, using MRI metrics, acute sleep deprivation was shown to induce changes in the cerebral cortical microstructure, resulting in reduced thickness in the parietal, insular, sensorimotor, and medial parietal cortices after a full night of sleep deprivation [40]. Secondly, another study demonstrated decreased thickness in the bilateral medial parietal cortices following sleep deprivation [41]. Thirdly, findings from another study revealed diminished volumes in the polar, superior, middle frontal gyri, temporal, and parietal lobes after 24 h of sleep deprivation [42]. These investigations collectively highlight the impact of sleep deprivation on microstructural cortical indices, particularly in areas associated with motor processing. Therefore, the current study speculates that sleep deprivation significantly affects high-intensity endurance performance, and the neural mechanism underlying this effect may be related to the attenuation of neural electrical activity in the central region and the left and right parietal lobes of the brain caused by sleep deprivation.

In this study, there are several limitations that could potentially affect the integrity and applicability of the conclusions. Future studies may consider incorporating voluntary exhaustion criteria alongside objective validation to enhance understanding of individual differences in endurance. Secondly, the experiment might not fully capture the long-term effects of sleep deprivation on neural activity and endurance performance. Prolonged sleep insufficiency may have more complex and sustained influences on the brain. It would be valuable to investigate the effects of sleep deprivation over extended periods to understand its cumulative impact. Additionally, controlling factors such as sleep quality and individual differences can be challenging in experimental design. These factors could potentially confound the relationship between sleep deprivation and exercise performance. It is crucial to address these variables to improve the validity of the findings. Furthermore, the study may not have considered other relevant factors such as lifestyle, environment, and other health-related variables that could influence the relationship between sleep and exercise. These factors might interact with sleep deprivation and impact exercise performance. Future research should take a comprehensive approach by considering these factors to gain a more holistic understanding of the impact of sleep deprivation on exercise performance.

## Conclusion

This study demonstrates that sleep deprivation significantly reduces high-intensity endurance performance through changes in brain neural activity and alterations in network connectivity. The observed reductions in δ and θ frequency band rhythms in central and parietal regions underscore the disruption of critical brain functions related to motor control and endurance. Additionally, the decreased efficiency of information transmission within δ band networks suggests that cognitive and motor processes are adversely affected, further compromising endurance performance.

## Data Availability

The datasets used and/or analysed during the current study are available from the corresponding author on reasonable request.

## Abbreviations

EEG: Electroencephalography
PLV: Phase-Locking Value
FFT: Fast Fourier transform

## References

[1] Ralbovska DC, Zavis M, Olah M. PSYCHOLOGICAL AND PHYSICAL CONSEQUENCES OF EMERGENCY SITUATIONS IN MEMBERS OF INTEGRATED RESCUE SYSTEM. Pakistan J Soc Educ Lang (PJSEL). 2019;5(1):65–72.

[2] Ein N, et al. Physical and psychological challenges faced by military, medical and public safety personnel relief workers supporting natural disaster operations: a systematic review. Curr Psychol. 2024;43(2):1743–58.

[3] Agori-Iwe OO. Impact of Sleep Deprivation on cognitive demand, attention to Detail, and Mental Processing Power in firefighters during emergencies. National University; 2024.

[4] Marvin G, et al. Occupation-Induced fatigue and impacts on Emergency First responders: a systematic review. Int J Environ Res Public Health. 2023;20(22):7055. 37998287 10.3390/ijerph20227055 PMC10671419

[5] Zhao S, et al. The neurological effects of acute physical exhaustion on inhibitory function. Physiol Behav. 2024;284:114641. 39019134 10.1016/j.physbeh.2024.114641

[6] Morgan JA, Corrigan F, Baune BT. Effects of physical exercise on central nervous system functions: a review of brain region specific adaptations. J Mol Psychiatry. 2015;3:1–13. 26064521 10.1186/s40303-015-0010-8 PMC4461979

[7] Zoccoli G, Amici R. Sleep and autonomic nervous system. Curr Opin Physiol. 2020;15:128–33.

[8] Frost C, et al. The effects of sleep on firefighter occupational performance and health: a systematic review and call for action. Sleep Epidemiol. 2021;1:100014.

[9] Bradley C, et al. State-dependent effects of neural stimulation on brain function and cognition. Nat Rev Neurosci. 2022;23(8):459–75. 35577959 10.1038/s41583-022-00598-1

[10] Souissi W, et al. Partial sleep deprivation affects endurance performance and psychophysiological responses during 12-minute self-paced running exercise. Physiol Behav. 2020;227:113165. 32891607 10.1016/j.physbeh.2020.113165

[11] Lim J, Dinges DF. Sleep deprivation and vigilant attention. Ann N Y Acad Sci. 2008;1129(1):305–22. 18591490 10.1196/annals.1417.002

[12] Krause AJ, et al. The sleep-deprived human brain. Nat Rev Neurosci. 2017;18(7):404–18. 28515433 10.1038/nrn.2017.55 PMC6143346

[13] Curcio M, Bradke F. Axon regeneration in the central nervous system: facing the challenges from the inside. Annu Rev Cell Dev Biol. 2018;34:495–521. 30044649 10.1146/annurev-cellbio-100617-062508

[14] Weavil JC, Amann M. Corticospinal excitability during fatiguing whole body exercise. Prog Brain Res. 2018;240:219–46. 30390833 10.1016/bs.pbr.2018.07.011 PMC6363483

[15] Zhang Y, et al. Effects of acute-partial sleep deprivation on high-intensity exercise performance and cardiac autonomic activity in healthy adolescents. Sustainability. 2021;13(16):8769.

[16] Lyytikäinen K. *Recovery of rescuers from a 24-hour shift and its association with physical fitness.* 2013. 10.13075/ijomeh.1896.00720 28481376

[17] Allen NJ, Lyons DA. Glia as architects of central nervous system formation and function. Science. 2018;362(6411):181–5. 30309945 10.1126/science.aat0473 PMC6292669

[18] Han C et al. *Complexity analysis of EEG signals for fatigue driving based on sample entropy*. in. 2018 *11th international congress on image and signal processing, biomedical engineering and informatics (CISP-BMEI)*. 2018. IEEE.

[19] Sandler H, et al. Positive emotional experience: induced by vibroacoustic stimulation using a body monochord in patients with psychosomatic disorders: is associated with an increase in EEG-theta and a decrease in EEG-alpha power. Brain Topogr. 2016;29:524–38. 26936595 10.1007/s10548-016-0480-8

[20] Kamiński J, et al. Beta band oscillations engagement in human alertness process. Int J Psychophysiol. 2012;85(1):125–8. 22155528 10.1016/j.ijpsycho.2011.11.006

[21] Siclari F, et al. Dreaming in NREM sleep: a high-density EEG study of slow waves and spindles. J Neurosci. 2018;38(43):9175–85. 30201768 10.1523/JNEUROSCI.0855-18.2018 PMC6199409

[22] Snipes S, et al. The theta paradox: 4–8 hz EEG oscillations reflect both sleep pressure and cognitive control. J Neurosci. 2022;42(45):8569–86. 36202618 10.1523/JNEUROSCI.1063-22.2022 PMC9665934

[23] Berg-Sørensen K, Flyvbjerg H. Power spectrum analysis for optical tweezers. Rev Sci Instrum. 2004;75(3):594–612.

[24] Bastos AM, Schoffelen J-M. A tutorial review of functional connectivity analysis methods and their interpretational pitfalls. Front Syst Neurosci. 2016;9:175. 26778976 10.3389/fnsys.2015.00175 PMC4705224

[25] Nir Y, et al. Selective neuronal lapses precede human cognitive lapses following sleep deprivation. Nat Med. 2017;23(12):1474–80. 29106402 10.1038/nm.4433 PMC5720899

[26] Song T, et al. Total sleep deprivation triggers greater activation in the parietal brain in the visual working memory updating processes: an event-related potentials study. Front NeuroSci. 2022;16:736437. 35368284 10.3389/fnins.2022.736437 PMC8966886

[27] Bender B. *Sleep Deprivation and the Health of Firefighters.* 2018.

[28] Hirshkowitz M, et al. National Sleep Foundation’s sleep time duration recommendations: methodology and results summary. Sleep Health. 2015;1(1):40–3. 29073412 10.1016/j.sleh.2014.12.010

[29] Itagi ABH, et al. Physical exhaustion induced variations in event-related potentials and cognitive task performance in young adults. Annals Neurosciences. 2019;25(4):299–304. 10.1159/000487845 PMC6470344 31000970

[30] Wang Z, Tong Y, Heng X. Phase-locking value based graph convolutional neural networks for emotion recognition. IEEE Access. 2019;7:93711–22.

[31] Niso G, et al. HERMES: towards an integrated toolbox to characterize functional and effective brain connectivity. Neuroinformatics. 2013;11:405–34. 23812847 10.1007/s12021-013-9186-1

[32] Javaheripour N, et al. Functional brain alterations in acute sleep deprivation: an activation likelihood estimation meta-analysis. Sleep Med Rev. 2019;46:64–73. 31063939 10.1016/j.smrv.2019.03.008 PMC7279069

[33] Valomon A, et al. Effects of COMT genotype and tolcapone on lapses of sustained attention after sleep deprivation in healthy young men. Neuropsychopharmacology. 2018;43(7):1599–607. 29472644 10.1038/s41386-018-0018-8 PMC5983551

[34] Tong F. Primary visual cortex and visual awareness. Nat Rev Neurosci. 2003;4(3):219–29. 12612634 10.1038/nrn1055

[35] Sharma A, et al. Sleep Deprivation-Induced blood-brain barrier breakdown and brain dysfunction are exacerbated by size-related exposure to Ag and Cu nanoparticles. Neuroprotective effects of a 5-HT3 receptor antagonist Ondansetron. Mol Neurobiol. 2015;52(2):867–81. 26133300 10.1007/s12035-015-9236-9

[36] Guo F, et al. Brain source imaging based on movement-related cortical potentials induced by fatigue during self-paced handgrip contractions. NeuroReport. 2020;31(4):300–4. 31895748 10.1097/WNR.0000000000001395

[37] Guo F, et al. Movement-related cortical potentials during muscle fatigue induced by upper limb submaximal isometric contractions. NeuroReport. 2014;25(14):1136–43. 25089802 10.1097/WNR.0000000000000242

[38] Hadjidimitrakis K, et al. Mixed spatial and Movement representations in the primate posterior parietal cortex. Front Neural Circuits. 2019;13:15. 30914925 10.3389/fncir.2019.00015 PMC6421332

[39] Jiang Z, et al. Strengthened functional connectivity in the brain during muscle fatigue. NeuroImage. 2012;60(1):728–37. 22197785 10.1016/j.neuroimage.2011.12.013 PMC3288326

[40] Dai X-J, et al. Plasticity and susceptibility of brain morphometry alterations to insufficient sleep. Front Psychiatry. 2018;9:266. 29997530 10.3389/fpsyt.2018.00266 PMC6030367

[41] Sun J, et al. Alteration of brain gray matter density after 24 h of sleep deprivation in healthy adults. Front NeuroSci. 2020;14:754. 32903801 10.3389/fnins.2020.00754 PMC7438917

[42] Elvsåshagen T, et al. Evidence for cortical structural plasticity in humans after a day of waking and sleep deprivation. NeuroImage. 2017;156:214–23. 28526620 10.1016/j.neuroimage.2017.05.027

